# The impact of migraine and probable migraine on productivity loss in Korea: A cross-sectional online survey

**DOI:** 10.1371/journal.pone.0277905

**Published:** 2022-11-28

**Authors:** Yejin Kim, Sola Han, Hae Sun Suh

**Affiliations:** 1 Department of Regulatory Science, Graduate School, Kyung Hee University, South Korea; 2 Institute of Regulatory Innovation through Science, Kyung Hee University, South Korea; 3 Health Outcomes Division, The University of Texas at Austin College of Pharmacy, Austin, Texas, United States of America; 4 College of Pharmacy, Kyung Hee University, Seoul, South Korea; Dokkyo Medical University: Dokkyo Ika Daigaku, JAPAN

## Abstract

Migraine is an enormous burden on society, but relevant studies are limited. The population of interest of this study was migraine or probable migraine (PM) in Korea. In this population, we aimed to assess the productivity loss through the level of severity defined by monthly migraine days (MMD) and analgesic frequency and to estimate costs and associated factors of productivity loss. We conducted an online survey of adults with migraine symptoms. We defined migraine and PM using the modified International Classification of Headache Disorders, second edition. Severity level was defined by subgroups of MMD (0–3, 4–14, and ≥15 days) and analgesic frequency (0, 1, 2, 3, and ≥4 per week). Productivity loss was assessed using the Work Productivity and Activity Impairment questionnaire and consisted of absenteeism, presenteeism, overall work productivity loss, and activity impairment. The costs of productivity loss due to absenteeism and presenteeism were calculated in 2020 USD. We used negative binomial regression to identify the factors associated with the costs of productivity loss. We identified 362 respondents with migraine or PM. Mean age was 41.7 years, 75.7% were female (N = 274), and 73.2% (N = 265) were employed. On average, productivity losses due to absenteeism and presenteeism were 8.1% and 39.7%, respectively. As MMD increased, there was a trend toward increased activity impairment, presenteeism, and overall work productivity loss. The mean overall productivity loss cost was USD 44.61 per person per day. Duration of migraine attacks was significantly associated with higher absenteeism costs. The results of this study indicate that the higher the MMD, the greater the productivity loss in patients with migraine or PM in Korea. We also found that patients with low-frequency migraine and PM experienced substantial productivity loss. This study provides comprehensive evidence of the burden of migraine in Korea using a representative sample.

## Introduction

Migraine is a very common and often disabling neurological disease, which places an enormous burden on individuals and society. In addition, headache-related disability is on the rise in Korea [1]. Furthermore, a recent Korean population-based study found that the disease burden of migraine patients who had received prophylactic treatments was significantly higher than those who had never received it [2]. Therefore, understanding the economic impact of migraine is increasingly important [3, 4]. Migraine was the second leading cause of years lived with disability (YLD), according to a 2016 study by the Global Burden of Disease [5]. A recent literature review found an apparent increase in the prevalence of migraine compared to the previous review in 2007 [6]. In addition, the prevalence and YLD rates of migraine were highest during the more productive years of life, from ages 35 to 39. Similarly, in a recent Korean study on both migraine and probable migraine (PM), the prevalence was highest in patients who were aged 30–39 and 40–49 years [7]. Despite the high prevalence and significance of migraine-related disability, there is limited information on the economic burden of migraine or migraine disability and productivity losses in East Asia, including South Korea [8].

Several studies conducted in Europe have found that migraine causes a substantial economic burden, including loss of productivity [9–11]. Furthermore, Doane et al. and Sumelahti et al. reported a tendency toward greater loss of productivity with increased migraine frequency [9, 10]. In Korea, Kim et al. confirmed the significant burden of migraine patients in the country [12]. However, studies on the relationship between the burden of migraine and migraine frequency are scarce in Korea.

Many patients who suffer from headaches satisfy the criteria for PM, but they haven’t been diagnosed with migraine [13]. Most of those diagnosed with PM did not meet the criterion of headache duration [13–15]. In Asia, PM has been reported as a common primary headache disorder with a prevalence of 6.2%–11.5% [13, 16]. PM is also known to be associated with disability and poor quality of life [13]. However, studies on disability and quality of life due to PM are scarce. Similarly, most migraine patients experience ≤3 monthly migraine days (MMD), but the migraine burden in this population is unknown [10]. If the burden of PM can be estimated, it would be meaningful in that it may be possible to estimate the comprehensive disease burden of migraine.

This study aims to estimate costs and associated factors of productivity loss in patients with migraine or PM in Korea, and to assess productivity loss according to the level of severity defined by MMD and the frequency of analgesic use.

## Materials and methods

### Recruitment and inclusion criteria

**Fig 1:**
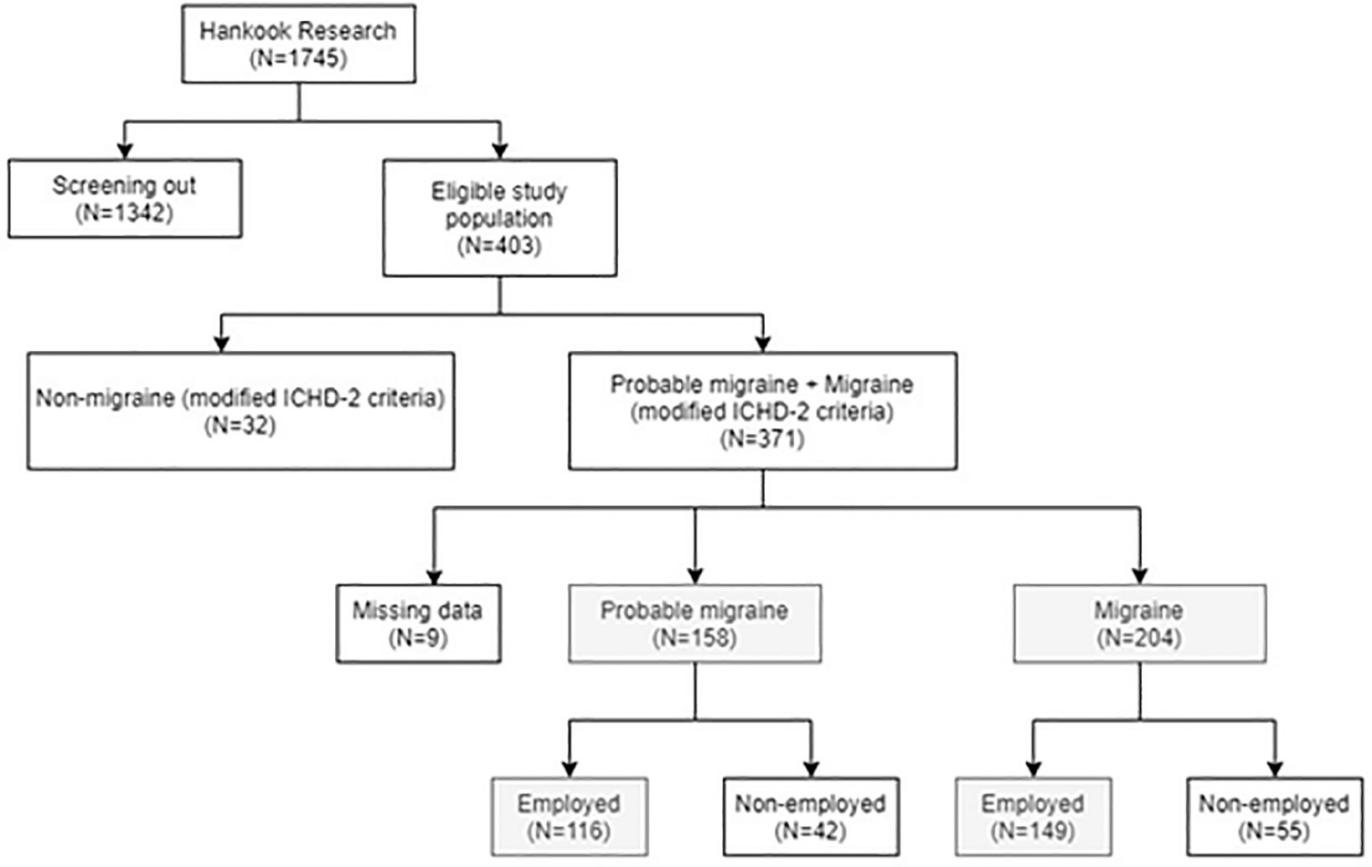
Flow chart of study sample selection. Abbreviations: ICHD-2, International Criteria of Headache Disorders 2nd edition.

The online survey was conducted from October 23 to November 2, 2020, in Korea by Hankook Research, a healthcare research company. The respondents were sampled using email from the Hankook Research panel members. The inclusion criteria were: (1) 18–69 years old; (2) living in Seoul, a metropolitan area, or in one of five major cities; (3) had experienced a migraine that interfered with daily life within the past 3 months; (4) had experienced periodic or recurrent migraines within the past 3 months; and (5) had fulfilled at least one modified International Classification of Headache Disorders, second edition (ICHD-2) criterion. There were 1745 respondents who satisfied at least one inclusion criterion, and among them, 403 satisfied all criteria. We defined migraine and PM by using criterion 1–3 of the modified ICHD-2. To conduct the valid survey, we referred to previous studies [17, 18] on the US 2004 AMPP Study using the ANS/AMPP diagnostic module. The AMS/AMPP diagnostic module has demonstrated sensitivity of 100% and specificity of 82.3% in a validation study between a population sample of clinically diagnosed migraine patients and controls with tension-type and other types of headaches [18]. No significant changes occurred between ICHD-2 and ICHD-3 criteria related to the criteria used in the studies [19]. Moreover, for the structured questionnaire, we had several face-to-face discussions with five prominent Korean neurologists experienced in migraine research. Based on the expert opinions, we revised the questionnaire to be clear and easily understandable. Respondents satisfied criterion 1 if they experienced one or more of the following associated with headache: moderate-to-severe intensity, unilateral location, pulsatile/throbbing pain, or aggravation by routine physical activity. Respondents satisfied criterion 2 if they experienced one or more of the following symptoms associated with headache: nausea or vomiting, photophobia and phonophobia, or visual or sensory aura. Respondents satisfied criterion 3 if they experienced headache lasting for 4–72 hours. If respondents met all three criteria, they were defined as migraine, and if respondents met two of the three criteria, they were defined as PM (Fig 1).

Prior to enrollment, informed written consent was obtained from all respondents. The study was approved by the Institutional Review Board of Pusan National University (PNU IRB/2020_142_HR).

### Measures

We measured demographic and clinical characteristics, including age, gender, education level, marital status, employment status, monthly household income, and level of headache severity. The level of severity was defined by subgroups of MMD (i.e., 0–3, 4–14, and ≥15 days) and the frequency of analgesic use (i.e., 0, 1, 2, 3, and ≥4 per week). The recall period for each question was 3 months.

The Work Productivity and Activity Impairment (WPAI) questionnaire is a validated self-administered instrument used to assess the impact of disease on productivity. It consists of four metrics: absenteeism (i.e., the percentage of work time missed because of one’s health in the past 7 days), presenteeism (i.e., the percentage of impairment experienced while at work in the past 7 days because of one’s health), overall work productivity loss (i.e., the overall work impairment measured by combining absenteeism and presenteeism), and activity impairment (i.e., the percentage of impairment in daily activities due to one’s health in the past 7 days) [20]. Absenteeism, presenteeism, and overall work productivity loss were calculated only for employed (i.e., full-time, part-time, or self-employed) respondents, because only these respondents possessed data on daily wages. A total of 265 respondents reported that they were employed (Fig 1). The effects of migraine on work productivity and daily activities were assessed using the Korean version of the validated WPAI questionnaire, modified for migraine assessment.

Costs of productivity loss due to absenteeism and presenteeism were calculated by integrating information from the WPAI questionnaire and hourly wage rates from the Survey Report on Labor Conditions by Employment Type, 2020 using the human capital method. For each employed respondent, hours lost due to absenteeism or presenteeism were multiplied by the average daily wage to estimate the total daily absenteeism, presenteeism, and overall work productivity loss costs. All costs were measured in Korean won (KRW) and converted to US dollars (USD) using the 2020 exchange rate (1USD = 1086.3 KRW).

### Sensitivity analysis

We performed a sensitivity analysis of the productivity loss cost analyses. For this sensitivity analysis, only patients defined with migraine were included.

### Statistical analyses

For the descriptive statistics, we used counts and percentages for categorical variables, and means and standard deviations for continuous variables. Chi-square tests were used for categorical variables and one-way analysis of variance for continuous variables.

In the analysis of the impact of MMD and the frequency of analgesic use on WPAI, if the normality assumption was violated, the non-parametric Kruskal-Wallis test was used to detect any statistically significant differences between MMD and frequency of analgesic use subgroups (with *p*<0.05). When significant differences were observed, the Bonferroni post-hoc test was used for paired comparisons between MMD and frequency of analgesic use subgroups in which *p*<0.017 and *p*<0.0083, respectively, were judged as statistically significant.

As the outcome variables are over-dispersed, we used negative binomial regression to identify the factors associated with productivity loss costs. Gender, MMD, frequency of analgesic use, and duration of migraine attacks were considered factors in this analysis.

All analyses were performed using IBM SPSS Statistics software (version 22.0 for Windows, IBM Corp., Armonk, NY, USA).

## Results

### Demographic and clinical characteristics

The number of respondents who were defined as migraine or PM was 371. The final sample size comprised of 362 migraine or PM patients, which did not include any missing values (Fig 1). The respondents provided their demographic characteristics, such as age, gender, education level, marital status, employment status, and monthly household income. Furthermore, they provided migraine-specific characteristics, including MMD and the frequency of analgesic use. The demographics of subgroups of MMD are presented in Table 1. The demographics of frequency of analgesic use subgroups are presented in S1 Table. The total number of respondents was 362, with a mean age of 41.7 years. In addition, the category with the largest number of patients was the 40–49 years of age. The majority used analgesics at least once a week and were mainly female across all groups (> 70%). Seventy-three percent of the respondents were employed at the time of the survey.

**Table 1:** Demographics of the MMD subgroups.

Variables	Probable migraine + Migraine: N, %								
	Overall		Monthly Migraine Days						
			≤3		4–14		≥15		*p*
	362		224		122		16		
Age (years) (mean (SD))	41.7	(11.8)	41.8	(12.0)	41.9	(11.6)	38.1	(9.8)	*0*.*46*
Age group (years)									
18–29	60	16.6%	40	17.9%	16	13.1%	4	25.0%	*0*.*79*
30–39	86	23.8%	49	21.9%	33	27.0%	4	25.0%	
40–49	131	36.2%	82	36.6%	43	35.2%	6	37.5%	
50–59	51	14.1%	30	13.4%	19	15.6%	2	12.5%	
60–69	34	9.4%	23	10.3%	11	9.0%	0	0.0%	
Gender									
Male	88	24.3%	50	22.3%	35	28.7%	3	18.8%	*0*.*36*
Female	274	75.7%	174	77.7%	87	71.3%	13	81.3%	
Education level									
≤High school graduation	121	33.4%	65	29.0%	51	41.8%	5	31.3%	*0*.*05*
≥College degree	241	66.6%	159	71.0%	71	58.2%	11	68.8%	
Monthly household income (USD)									
≤ 2,752	86	23.8%	46	20.5%	32	26.2%	8	50.0%	*0*.*05*
2,762–4,594	129	35.6%	88	39.3%	36	29.5%	5	31.3%	
4,603–6,435	87	24.0%	56	25.0%	31	25.4%	0	0.0%	
≥ 6,444	60	16.6%	34	15.2%	23	18.9%	3	18.8%	
Employment									
Employed	265	73.2%	167	74.6%	89	73.0%	9	56.3%	*0*.*28*
Non-employed	97	26.8%	57	25.4%	33	27.0%	7	43.8%	

Abbreviations: SD, Standard deviation.

Data are N(%) values. The exchange rate of Korean won to the US dollar was 1086.3 Korean won/US dollar in 2020. *≤High school graduation* means *‘elementary*, *middle*, *and high school graduation’*, and *≥College degree* means *‘having college degree or over’*.

### Work Productivity and Activity Impairment (WPAI)

On average, the employed respondents (N = 265) had lost 8.1% and 44.3% of productive time due to absenteeism and presenteeism, respectively. This resulted in 47.9% of overall work impairment, which includes absenteeism and presenteeism, and 47.9% of activity impairment. The results of activity impairment were derived from the whole population (N = 362) including the unemployed.

**Fig 2:**
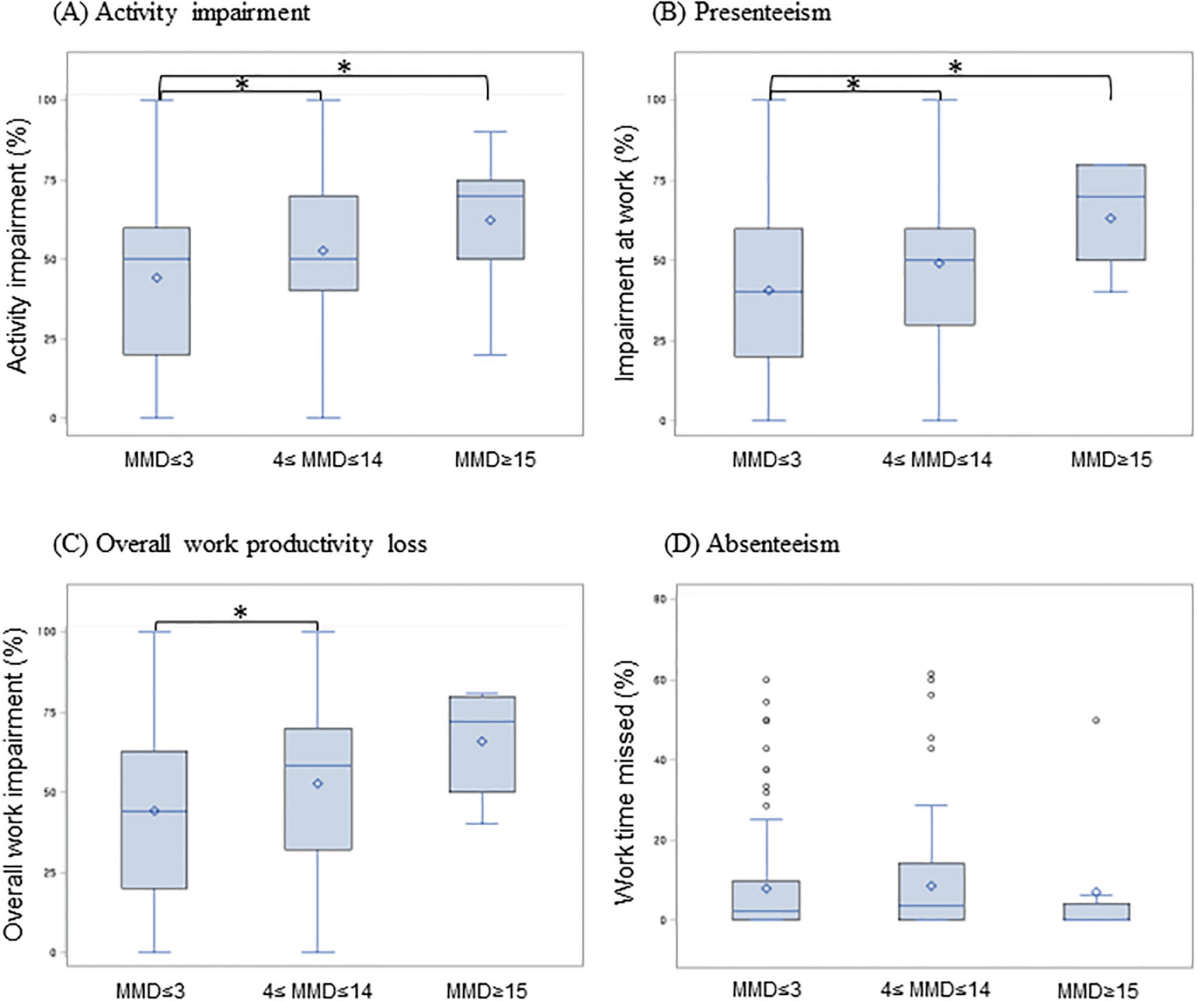
The impact of monthly migraine days on Work Productivity and Activity Impairment during previous 1-week. (A) Activity impairment (N = 362) (B) Presenteeism (N = 265) (C) Overall work productivity loss (N = 265) (D) Absenteeism (N = 265) *p<0.017.

**Fig 3:**
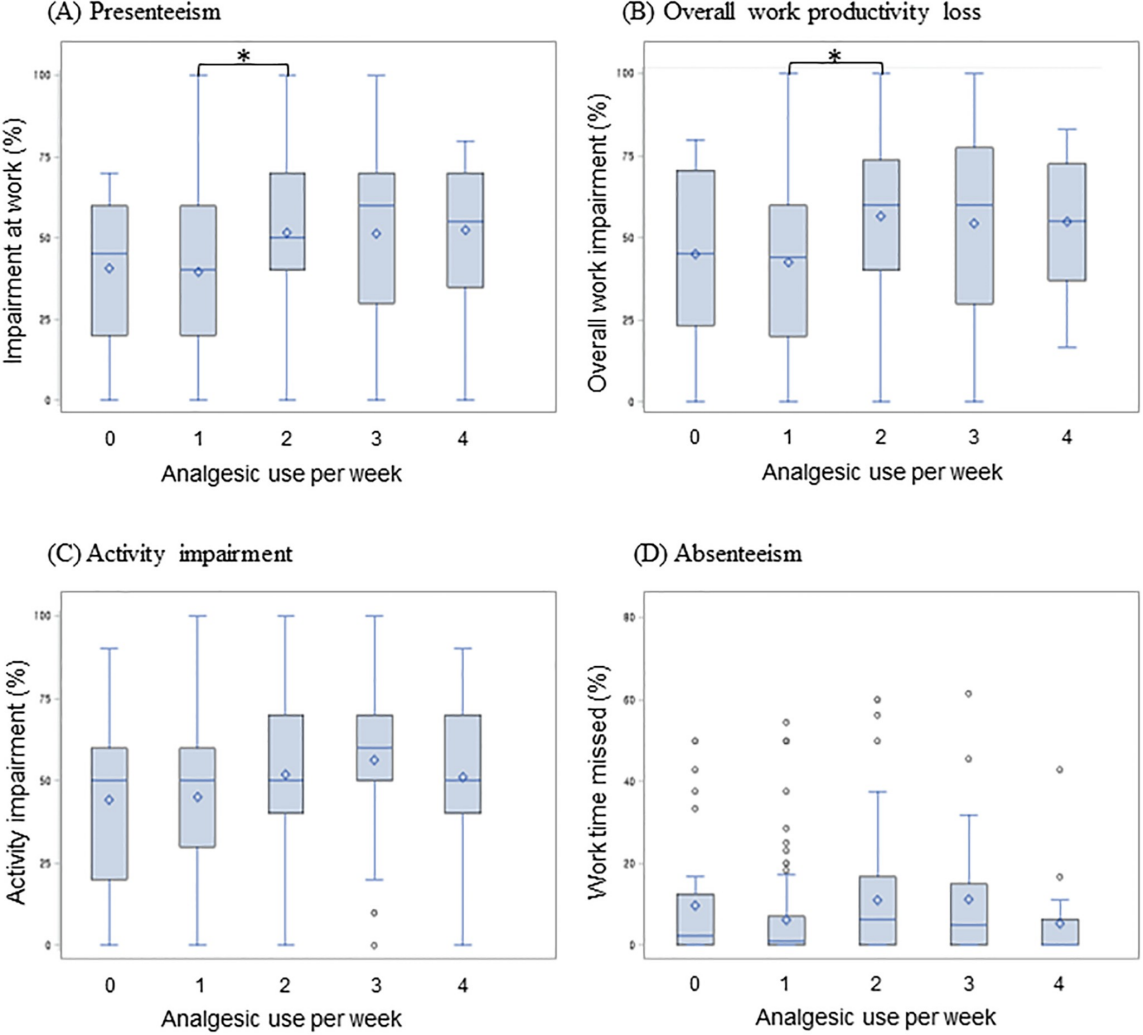
The impact of frequency of analgesic use on Work Productivity and Activity Impairment during previous 1-week. (A) Presenteeism (N = 265) (B) Overall work productivity loss (N = 265) (C) Activity impairment (N = 362) (D) Absenteeism (N = 265) *p<0.0083.

The productivity losses (i.e., activity impairment, presenteeism, overall work productivity loss, and absenteeism) by MMD subgroups and frequency of analgesic use subgroups are reported in Figs 2 and 3, respectively. As shown in Fig 2, the activity impairment and presenteeism were increased with the MMD, and 4–14 MMD subgroups and the ≥15 MMD subgroup showed significantly higher impairment compared to the ≤3 MMD subgroup (*p*<0.017). The overall work productivity loss was increased by MMD, but only the 4–14 MMD subgroup showed significantly higher impairment compared to the ≤3 MMD subgroup. However, absenteeism did not significantly differ between MMD subgroups (Fig 2). Unexpectedly, productivity losses in frequency of analgesic use subgroups showed generally similar trends between the groups (Fig 3).

**Fig 4:**
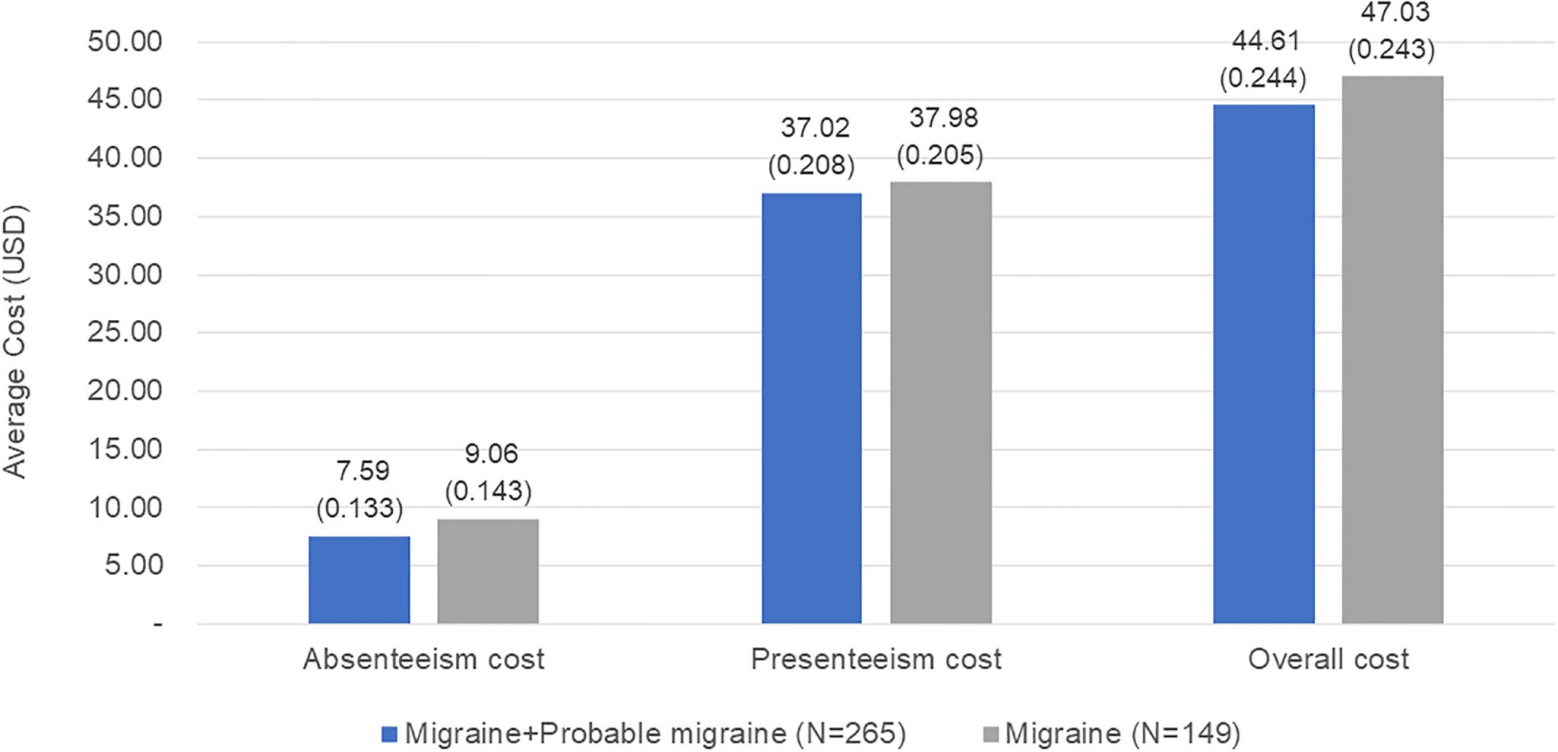
Mean of daily productivity loss cost. The exchange rate of Korean won to the US dollar was 1086.3 Korean won/US dollar in 2020. The mean and standard deviation of each cost is presented in the graph. Migraine is defined as respondents who met all three modified ICHD-2 criteria. Probable migraine is defined as respondents who met two of three modified ICHD-2 criteria.

Fig 4 shows the cost of productivity loss. In the ‘migraine and probable migraine’ group (N = 265), the mean overall cost of productivity loss was USD 44.61 per person per day. Furthermore, approximately 83% of these costs were due to presenteeism (USD 37.02). Costs of productivity loss were greater in the ‘migraine’ group than those in the ‘migraine and probable migraine’ group. In the ‘migraine’ group (N = 149), the mean overall cost of productivity loss was USD 47.03 per patient per day (Fig 4).

### Regression

In negative binomial regression, the exponential of the coefficient represents the ratio of each cost of productivity loss between the groups (Table 2). For example, a ratio of 0.555 in the once-a-week analgesic group indicates that absenteeism cost is 44.5% less than the non-analgesic-use group. Being female decreased expected absenteeism cost by about 30% (95% CI: 0.524, 0.911) compared to being male. MMD and frequency of analgesic use were not significant predictors of presenteeism cost and overall cost.

**Table 2:** Multivariate analyses about predictors for productivity loss cost among Korean migraine and probable migraine respondents.

Cost outcome variable	Associated variable		Coefficient [95% CI]	p-value
**Absenteeism Cost**	Gender	*Male*	Reference	***0*.*009***
		*Female*	0.691 [0.52, 0.91]	
	MMD	*≤3*	Reference	
		*4–14*	0.790 [0.59, 1.05]	0.109
		*≥15*	0.807 [0.39, 1.68]	0.567
	Frequency of analgesic use	*Zero*	Reference	
		*Once a week*	0.555 [0.36, 0.85]	***0*.*006***
		*Twice a week*	0.984 [0.62, 1.56]	0.947
		*Three times a week*	1.157 [0.66, 2.04]	0.615
		*≥four times a week*	0.396 [0.21, 0.76]	***0*.*006***
	Duration of migraine attacks		1.007 [1.00, 1.01]	***0*.*027***
**Presenteeism Cost**	Gender	*Male*	Reference	0.342
		*Female*	1.166 [0.85, 1.60]	
	MMD	*≤3*	Reference	
		*4–14*	1.284 [0.93, 1.77]	0.127
		*≥15*	1.738 [0.76, 3.99]	0.193
	Frequency of analgesic use	*Zero*	Reference	
		*Once a week*	1.006 [0.63, 1.61]	0.981
		*Twice a week*	1.359 [0.81, 2.29]	0.247
		*Three times a week*	1.101 [0.59, 2.06]	0.764
		*≥four times a week*	1.267 [0.58, 2.77]	0.553
	Duration of migraine attacks		0.997 [0.99, 1.00]	0.284
**Overall Cost**	Gender	*Male*	Reference	
		*Female*	1.079 [0.789, 1.48]	0.638
	MMD	*≤3*	Reference	
		*4–14*	1.197 [0.87, 1.66]	0.278
		*≥15*	1.425 [0.61, 3.32]	0.411
	Frequency of analgesic use	*Zero*	Reference	
		*Once a week*	0.923 [0.58, 1.48]	0.738
		*Twice a week*	1.266 [0.76, 2.13]	0.371
		*Three times a week*	1.072 [0.57, 2.01]	0.828
		*≥four times a week*	1.055 [0.48, 2.32]	0.894
	Duration of migraine attacks		0.999 [0.99, 1.01]	0.719

Abbreviations: MMD, Monthly Migraine Days; CI, Confidence interval.

For presenteeism and overall cost, there were no statistically significant differences in the costs for all covariates.

## Discussion

The purpose of this study was to measure productivity loss associated with migraine and PM in Korea. This study found that as MMD increased, activity impairment, presenteeism, and overall work productivity loss tended to increase. Productivity losses by the frequency of analgesic use showed a generally similar trend between the subgroups.

We included respondents who were considered to have migraine or PM by using a representative sample in Korea. In three previous studies, the demographic characteristics of respondents, in terms of age and the women-to-men ratio, were similar to those in this study [12–14]. For the PM-to-migraine prevalence ratio, it is difficult to compare findings between studies, because all the studies have different definitions, sampling methods, and cultural backgrounds. In previous studies, the PM-to-migraine prevalence ratio ranged between 0.33 and 2.00 [13, 21, 22]. Kim et al. reported that the PM-to-migraine prevalence ratio was 1.92, and an intermediate value of 0.89 was reported in a previous French study [13, 14]. In the current study, the PM-to-migraine prevalence ratio is 0.77, which is similar to the study from France [14]. The variation between the results of the previous Korean study and the current study seems to be due to the different sampling methods, designs, and questionnaires.

Martelletti et al. reported absenteeism (14.4%) and presenteeism (35.5%) among Italian migraine patients with ≥4 monthly headache days [23]. Kikui at al. reported absenteeism (6.4%) and presenteeism (40.2%) among Japanese migraine patients with ≥4 MMD [24]. In this study, absenteeism (8.1%) was lower and presenteeism (39.7%) was higher than those of the Italian study, but similar to those of the Japanese study [23, 24]. ’Effort’ has always been seen as a virtue in Confucian culture, which persists in Asia. As a result of additional cultural pressures, Asian presenteeism may be higher than in the West [25]. A similar study conducted in the Asian country reported that individuals with migraine are difficult to be understood their pain at work and feel guilty about putting a burden on their bosses and colleagues [26]. According to these previous studies and the expert opinion of Korean neurologists, this difference could be due to cultural characteristics. Furthermore, in this study, we found that there were tolerant participants who took lots of analgesics to go to work but were less productive at work. It is known that awareness of migraine is low, therefore over-the-counter (OTC) medication use is frequent in East Asia [8]. According to a population-based study in Korea, only 24.4% of migraine patients had consulted a physician, and more than 90% of them were using OTC medications [27]. This suggests caution might be needed in medication overuse and headache occurred by overuse.

We observed a correlation between MMD and loss of productivity. In addition, we also assessed productivity loss by the frequency of analgesic use, but there was no trend of significant variation. We used the frequency of analgesic use because it reflects the frequency of headaches and migraines that were clinically meaningful and severe enough to require analgesics based on the clinical expert opinion of Korean neurologists.

Although several studies have assessed productivity loss costs, it is difficult to compare studies due to differences in study methods, survey instruments, and wage rates. Despite these variations, several studies have shown that presenteeism is the most influential driver of overall productivity loss costs [23, 24, 28, 29]. Martelletti et al. reported that 74% of the overall productivity loss costs were attributed to presenteeism [23]. In Asian countries, productivity loss costs due to presenteeism accounted for more than 80%, 78% and 63% of the overall productivity loss costs in Japan, Malaysia and the Philippines, respectively [24, 28, 29]. Similarly, in the current study, over 80% of overall productivity loss costs were due to presenteeism. The overall productivity loss cost per person per day for all employed respondents was USD 44.61 for the ‘migraine and PM’ group, compared to USD 47.03 for the ‘migraine’ group. However, it was difficult to find the exact reason why the burden of presenteeism was bigger than that of absenteeism in any of these studies. The reason might be whether to go to work is quite subjective, and several external factors such as culture and living standards influence this, but further research is still needed [30, 31].

We found that being female was associated with 0.309 times less absenteeism cost compared to being male. Similarly, Kikui et al. reported that being female was associated with 0.53 times less absenteeism cost compared to being male [24]. In addition, a longer duration of the migraine attacks was associated with 1.007 (95% CI: 1.001, 1.013) times more absenteeism cost. Except for Kikui et al., there is a lack of research on covariates associated with migraine-related productivity loss, therefore, it is difficult to compare. Further research in this area is thus needed.

This study has some limitations. First, most respondents had ≤3 MMD, and the number of respondents from the most severe group (e.g., ≥15 MMD) was small (4.4%). Compared with other studies, the number of patients with ≥15 MMD was smaller in our study [9, 12, 24]. Because of this, it was difficult to identify the tendency of absenteeism accurately. Nevertheless, the more severe the migraine, the higher the presenteeism tends to be. This means that individuals with Chronic migraine have become so used to this headache pain that they live with it. In addition, many factors are related to whether not to go to work, one of which is migraine-related stigma [32, 33]. However, we found the productivity loss was highest in the ≥15 MMD subgroup, similar to what previous studies reported [9, 12, 24]. Second, because we could not access the medical records of respondents, we defined respondents with migraine as those exhibiting symptoms of migraine by using the modified ICHD-2 criteria that were revised to be clear and easily understandable based on the expert opinions of Korean neurologists.

This study also has several advantages. Despite the higher prevalence of ≤3 MMD and PM, evidence on the burden of disease in these populations is scarce [10, 12, 13, 16]. Therefore, this study is significant in that it comprehensively assessed productivity loss in patients with migraine, including patients with ≤3 MMD and PM that were rarely reported in previous studies. In addition, to the best of our knowledge, this is the first study to assess productivity loss by level of severity defined by MMD, analgesic frequency, estimated productivity loss costs, and associated factors in Korea.

## Conclusions

This study provides comprehensive evidence of the burden of migraine in Korea using a representative sample. The results of this study indicate that the higher the severity of migraine, the greater the productivity loss of migraine in Korea. In addition, low-frequency migraine and PM also cause substantial productivity loss. Finally, the findings of this study suggest there is a need for migraine prevention strategies to reduce productivity loss and societal burden.

## Supporting information

S1 Table Demographics of analgesic use subgroups. Abbreviations: SD, Standard deviation. Data are N(%) values. The exchange rate of Korean won to the US dollar was 1086.3 Korean won/US dollar in 2020. ≤High school graduation means ‘elementary, middle, and high school graduation’, and ≥College degree means ‘having college degree or over’.
(DOCX)
