# Supplementary material for: The impact of migraine and probable migraine on productivity loss in Korea: A cross-sectional online survey
Source: PLoS One. 2022 Nov 28;17(11):e0277905. doi: 10.1371/journal.pone.0277905 (PMC9704660; doi:10.1371/journal.pone.0277905)
Supplement: S1 Table — Abbreviations: SD, Standard deviation. Data are N(%) values. The exchange rate of Korean won to the US dollar was 1086.3 Korean won/US dollar in 2020. ≤High school graduation means ‘elementary, middle, and high school graduation’, and ≥College degree means ‘having college degree or over’. (DOCX) [file pone.0277905.s001.docx]

S1 table. Demographics of analgesic use subgroups

| **Variables** |  |  | **Probable migraine + Migraine: N, %** | | | | | | | | | | |
| --- | --- | --- | --- | --- | --- | --- | --- | --- | --- | --- | --- | --- | --- |
|  | **Overall** | | **Analgesic use (days per week)** | | | | | | | | | | |
|  |  | | **0** | | **1** | | **2** | | **3** | | **≥4** | | ***p*** |
|  | **362** | | **55** | | **170** | | **82** | | **35** | | **20** | |  |
| Age (years) (mean (SD)) | 41.71 | (11.79) | 40.62 | (14.23) | 41.2 | (10.81) | 42.3 | (11.91) | 41.97 | (11.98) | 46.2 | (11.71) | *0.419* |
| Age group (years) |  |  |  |  |  |  |  |  |  |  |  |  |  |
| 18-29 | 60 | 16.6% | 17 | 30.9% | 22 | 12.9% | 13 | 15.9% | 5 | 14.3% | 3 | 15.0% | *0.18* |
| 30-39 | 86 | 23.8% | 7 | 12.7% | 49 | 28.8% | 19 | 23.2% | 9 | 25.7% | 2 | 10.0% |  |
| 40-49 | 131 | 36.2% | 18 | 32.7% | 64 | 37.6% | 28 | 34.1% | 12 | 34.3% | 9 | 45.0% |  |
| 50-59 | 51 | 14.1% | 5 | 9.1% | 24 | 14.1% | 13 | 15.9% | 6 | 17.1% | 3 | 15.0% |  |
| 60-69 | 34 | 9.4% | 8 | 14.5% | 11 | 6.5% | 9 | 11.0% | 3 | 8.6% | 3 | 15.0% |  |
| Gender |  |  |  |  |  |  |  |  |  |  |  |  |  |
| Male | 88 | 24.3% | 15 | 27.3% | 35 | 20.6% | 23 | 28.0% | 10 | 28.6% | 5 | 25.0% | *0.64* |
| Female | 274 | 75.7% | 40 | 72.7% | 135 | 79.4% | 59 | 72.0% | 25 | 71.4% | 15 | 75.0% |  |
| Education level |  |  |  |  |  |  |  |  |  |  |  |  |  |
| ≤High school graduation | 121 | 33.4% | 22 | 40.0% | 45 | 26.5% | 33 | 40.2% | 13 | 37.1% | 8 | 40.0% | *0.13* |
| ≥College degree | 241 | 66.6% | 33 | 60.0% | 125 | 73.5% | 49 | 59.8% | 22 | 62.9% | 12 | 60.0% |  |
| Monthly household income (USD) |  |  |  |  |  |  |  |  |  |  |  |  |  |
| ≤ 2,752 | 86 | 23.8% | 20 | 36.4% | 29 | 17.1% | 19 | 23.2% | 12 | 34.3% | 6 | 30.0% | *0.18* |
| 2,762-4,594 | 129 | 35.6% | 17 | 30.9% | 60 | 35.3% | 36 | 43.9% | 9 | 25.7% | 7 | 35.0% |  |
| 4,603-6,435 | 87 | 24.0% | 11 | 20.0% | 47 | 27.6% | 16 | 19.5% | 9 | 25.7% | 4 | 20.0% |  |
| ≥6444 | 60 | 16.6% | 7 | 12.7% | 34 | 20.0% | 11 | 13.4% | 5 | 14.3% | 3 | 15.0% |  |
| Employment |  |  |  |  |  |  |  |  |  |  |  |  |  |
| Employed | 265 | 73.2% | 32 | 58.2% | 133 | 78.2% | 61 | 74.4% | 23 | 65.7% | 16 | 80.0% | *0.04* |
| Non-employed | 97 | 26.8% | 23 | 41.8% | 37 | 21.8% | 21 | 25.6% | 12 | 34.3% | 4 | 20.0% |  |
| Abbreviations: SD, Standard deviation  Data are N(%) values. The exchange rate of Korean won to the US dollar was 1086.3 Korean won/US dollar in 2020. *≤High school graduation* means *‘elementary, middle, and high school graduation’*, and *≥College degree* means *‘having college degree or over’*. | | | | | | | | | | | | | |
